# Optimising the quantification of cytokines present at low concentrations in small human mucosal tissue samples using Luminex assays^☆^

**DOI:** 10.1016/j.jim.2013.04.009

**Published:** 2013-08-30

**Authors:** Emily Staples, Richard James Michael Ingram, John Christopher Atherton, Karen Robinson

**Affiliations:** Nottingham Digestive Diseases Biomedical Research Unit, Queen's Medical Centre, Nottingham, NG7 2UH, UK; Centre for Biomolecular Sciences, University of Nottingham, Nottingham, NG7 2RD, UK

**Keywords:** Luminex, Multiplex, Cytokine, Mucosal tissue, *Helicobacter pylori*

## Abstract

Sensitive measurement of multiple cytokine profiles from small mucosal tissue biopsies, for example human gastric biopsies obtained through an endoscope, is technically challenging. Multiplex methods such as Luminex assays offer an attractive solution but standard protocols are not available for tissue samples. We assessed the utility of three commercial Luminex kits (VersaMAP, Bio-Plex and MILLIPLEX) to measure interleukin-17A (IL-17) and interferon-gamma (IFNγ) concentrations in human gastric biopsies and we optimised preparation of mucosal samples for this application. First, we assessed the technical performance, limits of sensitivity and linear dynamic ranges for each kit. Next we spiked human gastric biopsies with recombinant IL-17 and IFNγ at a range of concentrations (1.5 to 1000 pg/mL) and assessed kit accuracy for spiked cytokine recovery and intra-assay precision. We also evaluated the impact of different tissue processing methods and extraction buffers on our results. Finally we assessed recovery of endogenous cytokines in unspiked samples. In terms of sensitivity, all of the kits performed well within the manufacturers' recommended standard curve ranges but the MILLIPLEX kit provided most consistent sensitivity for low cytokine concentrations. In the spiking experiments, the MILLIPLEX kit performed most consistently over the widest range of concentrations. For tissue processing, manual disruption provided significantly improved cytokine recovery over automated methods. Our selected kit and optimised protocol were further validated by measurement of relative cytokine levels in inflamed and uninflamed gastric mucosa using Luminex and real-time polymerase chain reaction. In summary, with proper optimisation Luminex kits (and for IL-17 and IFNγ the MILLIPLEX kit in particular) can be used for the sensitive detection of cytokines in mucosal biopsies. Our results should help other researchers seeking to quantify multiple low concentration cytokines in small tissue samples.

## Highlights

• Challenge: To evaluate in vivo cytokine profiles using small mucosal tissue samples
• Solution: Low volume multiplex assays and optimised sample preparation
• We compared low concentration IL-17 and IFNγ quantification by three Luminex kits.
• MILLIPLEX was most consistent for sensitivity and accurate spiked cytokine recovery.
• Manual tissue processing and PBS-based extraction buffers were superior.

## 1 Introduction

Assessing cytokine profiles in small tissue biopsies presents a significant technical challenge, particularly the quantification of multiple cytokines when some are present at low concentrations. Multiplex methods using Luminex technology may offer an attractive solution. However these are often developed using soluble materials such as sera or cell culture supernatants spiked with recombinant cytokines and standard protocols are not available for tissue samples. Luminex assays use multiple sets of polystyrene or paramagnetic beads or ‘microspheres’ — see Vignali (2000) and Houser (2012). Each set is fluorescently colour-coded to be identifiable on a dedicated flow cytometer or other platform and pre-coated with antibody to capture a specific cytokine or other analyte, around which a sandwich immunoassay is built. Different bead sets can be combined to enable simultaneous measurement of multiple cytokine concentrations in a single sample against standard curve preparations. These assays require substantially less sample than traditional enzyme-linked immunosorbent assays (ELISAs) – typically 25–50 μL for multiple analytes compared with 200 μL for a single analyte – yet may offer similar sensitivity to Luminex (Vignali, 2000; Biagini et al., 2004; Elshal and McCoy, 2006).

Our research concerns the characterisation of immune responses to the pathogen *Helicobacter pylori* (*Hp*) which are linked to peptic ulceration and gastric cancer development (Atherton, 2006; Robinson et al., 2008). The challenges are broadly similar in other fields, particularly for gastrointestinal mucosal researchers: how to study immune responses using methodology that better reflects cytokine levels in the mucosa in vivo. Endoscopic mucosal biopsies are small (typically around 5–10 mg) and concentrations of many of the cytokines of interest are low, so assay sensitivity and sample volume requirements are critical. Other investigators have used semi-quantitative methods including immunohistochemistry (Lindholm et al., 1998; Lehmann et al., 2002; Holck et al., 2003) and western blotting (Luzza et al., 2000; Tomita et al., 2001), or PCR-based methods to quantify cytokine mRNA which are not always fully reflected at the protein level (Luzza et al., 2001; Robinson et al., 2008; Serelli-Lee et al., 2012). Cytokines have been measured in gastric biopsy homogenates using ELISA (Yamaoka et al., 2001; Shimizu et al., 2004; Caruso et al., 2008; Robinson et al., 2008; Serelli-Lee et al., 2012), but additional volume is needed for each analyte assayed which may require sample dilution. Therefore the number of cytokines, particularly those present at low concentrations, that can be assayed using this method is limited. Another common approach is to culture gastric biopsies in vitro, with or without stimulation, and measure cytokine concentrations in culture supernatants (Crabtree et al., 1991; Bodger et al., 1997; Mizuno et al., 2005). However, these methods may alter the cytokine profile (Veldhoen et al., 2009). The cytokine concentrations in homogenates of gastric biopsies should more closely reflect those found in the gastric mucosa in vivo.

Luminex-based methods have been used to assess murine immune responses to *Hp* infection (Taylor et al., 2008) and vaccination (Taylor et al., 2007) in splenocyte culture supernatant and recently to quantify gastric cytokine concentrations in *Hp*-infected mice (Schumacher et al., 2012). A method to measure *Hp*-specific IgG in human saliva samples has also been developed, using Luminex beads conjugated with antigens including *Hp* whole cell sonicate and recombinant urease (Griffin et al., 2011). However, to our knowledge, Luminex assays have not been optimised for human gastrointestinal mucosal tissue samples, though were recently used to quantify interleukin-1β, interleukin-1 receptor antagonist, interleukin-6 and tumour necrosis factor-α in gastric tissue samples (Serelli-Lee et al., 2012). Careful kit selection and optimisation of tissue sample preparation in a limited volume of extraction buffer will theoretically facilitate cytokine detection in these samples.

Here we aim to systematically compare and contrast the accuracy and performance of several commercially available Luminex assays as well as different sample homogenisation protocols for quantification of cytokines in tissue biopsies. We purchased assays from three suppliers: Bio-Plex Pro (Bio-Rad Laboratories, CA, USA), MILLIPLEX MAP (Merck Millipore, Darmstadt, Germany) and VersaMAP (R&D Systems, MN, USA) with assays for interleukin-17A (IL-17) and interferon-gamma (IFNγ). This evaluation using cytokine spiked human gastric biopsies provides more widely relevant information on the technology's ability to quantify cytokines present at low concentrations in small tissue samples and optimisation of mucosal tissue preparation for this application. Finally we report on the suitability of our selected Luminex kit and optimised homogenisation protocol to detect endogenous cytokines in uninfected and *Hp*-infected clinical samples.

## 2 Materials and methods

### 2.1 Patients and samples

Patients attending for clinically-indicated routine upper gastrointestinal endoscopy at Queen's Medical Centre (Nottingham, UK) donated additional gastric mucosal biopsies for research. These were immediately snap frozen in liquid nitrogen and stored at − 80 °C. Patients were ineligible for inclusion in the study if they had previous gastric surgery, were regularly taking non-steroidal anti-inflammatory drugs (those taking regular aspirin for cardiovascular prophylaxis were not excluded), regular steroids or other immunosuppressive therapy, or had taken antibiotics in the preceding four weeks or proton pump inhibitors in the preceding two weeks. Written informed consent was obtained from all participants after the nature and possible consequences of the studies had been fully explained. Ethical approval was granted by the National Research Ethics Service East Midlands — Nottingham 2 Committee (08/H0408/195).

For the kit and tissue processing comparisons, seven patients (mean age ± standard deviation (SD) [range]; 51 ± 19 years [21–69]; two male, five female) each donated nine antral biopsies which were stored for up to 10 weeks until sample preparation. For evaluation of uninfected and *Hp*-infected tissue by Luminex cytokine assays, antral biopsies from a further 24 patients were used (51 ± 15 years [17–75]; 13 male, 11 female) of whom 18 were *Hp*+ and none of the six *Hp*− patients had evidence of gastric inflammation by histology. To determine mRNA expression we used antral biopsies from a further 41 consecutive patients (51 ± 15 years [29–81]; 17 male, 24 female) such that each transcript was evaluated in 18 *Hp*+ and 6 *Hp*− patients as complete data were not available for every patient. *Hp* status was assessed by biopsy urease test, culture, histology and IgG serology, with patients classified as infected if supported by at least three parameters and non-infected if all four parameters were negative with no history of previous eradication therapy.

### 2.2 Sample preparation methods

Single biopsies from each patient were individually thawed on ice then immediately disrupted in extraction buffer, either: (1) manually with a mini pellet pestle (Kimble Kontes, NJ, USA) for 2 min, (2) a proportion of those disrupted by pestle were further homogenised by 5–10 repeated passes through a 23 G needle and 1 mL syringe, or (3) automatically with a bead-basher (TissueLyser LT, QIAGEN, Hilden, Germany) using a single 5 mm stainless steel bead per sample at 50 Hz for 3 min. Extraction buffer comprised either: (A) RPMI-1640 (Sigma-Aldrich, MO, USA) supplemented with 10% (v/v) fetal calf serum (FCS, heat-inactivated, Sigma-Aldrich), (B) phosphate-buffered saline (PBS, pH 7.4, Dulbecco A, Oxoid, Basingstoke, UK), or (C) PBS supplemented with 2 mM Mg^2 +^ (Sigma-Aldrich) and benzonase endonuclease (at 25 U/mL, > 90% pure, Novagen, Darmstadt, Germany). Protease inhibitors (cOmplete mini [EDTA-free], Roche, Basel, Switzerland) were included in each extraction buffer. After disruption/homogenisation, all samples were incubated on ice for 5 min to allow sufficient time for viscosity reduction in endonuclease-supplemented samples. Finally, supernatants were obtained by centrifugation at 10,000 ×*g* for 10 min at 4 °C, spiked and split into aliquots as required (see below), and stored in Protein LoBind tubes (Eppendorf, Hamburg, Germany) at − 80 °C until analysis.

We also evaluated two commercial kits that extract proteins from tissue samples in accordance with the manufacturers' instructions (NucleoSpin TriPrep, Macherey-Nagel, Düren, Germany; RNA/DNA/Protein Purification Plus Kit, Norgen Biotek, ON, Canada) but found that the resulting protein samples interfered with Luminex assay function (data not shown).

### 2.3 Cytokine spiking of samples

To assess kit performance and accuracy, nine biopsies each from three patients were individually prepared using method (1) and extraction buffer (A). 50 μL of each of the resulting supernatants for each patient were combined (to give a total volume of 450 μL per patient), then split into three aliquots and spiked with 15 μL of known concentrations of both recombinant human IL-17 and IFNγ (eBioscience, CA, USA) diluted in extraction buffer (A). Cytokine spikes were at final concentrations of 0.0 (“unspiked”), 1.5, 6.0, 50.0, 100.0 and 1000.0 pg/mL. A single technical replicate was included in each run.

Biopsies from a further four patients were used to optimise processing methods and assess repeatability (intra-assay precision). Biopsies were processed using methods (1), (1) and (2), or (3) in 600 μL of PBS-based extraction buffer (B) or (C). Multiple pairs of biopsies from each patient were spiked prior to processing, either with recombinant human IL-17 and IFNγ (Merck Millipore) at a final concentration of 100.0 pg/mL in extraction buffer or with extraction buffer alone (“unspiked”). At least two technical replicates for each sample were included in each run. Cytokine recovery was adjusted for background cytokine concentrations from the unspiked samples and the different processing methods were compared. Repeatability was assessed using four technical replicates each from three of these samples, included at different positions on the same assay plate. The coefficient of variation (%CV) was calculated for each as [SD / mean] × 100.

### 2.4 Luminex cytokine assays and data analysis

Assays were run according to each manufacturer's instructions. The VersaMAP and Bio-Plex kits used non-magnetic beads (5.6 μm diameter) and the MILLIPLEX kit used paramagnetic beads (6.5 μm diameter). Filter plates and vacuum washing were used for all three kits for comparison. Standards were assayed in duplicate as provided by each manufacturer and standard curves extended down to < 1.0 pg/mL with additional steps. For subsequent assessment of endogenous cytokines in unspiked samples we used MILLIPLEX kits. Assays were run as per manufacturers' instructions with standards and samples in duplicate, overnight incubation with shaking at 4 °C (18 h, 750 rpm) and using a hand-held magnetic block for wash steps.

Data were acquired on a validated and calibrated Bio-Plex 200 system (Bio-Rad) and analysed with Bio-Plex Manager 6.0 software (Bio-Rad) with a detection target of 50 beads per region, low RP1 target for CAL2 calibration, and recommended doublet discriminator (DD) gates of 5000–25,000 for Bio-Plex and MILLIPLEX kits and 4300–10,000 for the VersaMAP kit. Standard, control and sample wells with bead counts < 37 were excluded as at least this number is required to minimise the potential impact of outlier beads on median fluorescence intensity (MFI). We excluded from the standard curve any points with %CV < 25% and those with accuracy outside of 80–120% of expected were excluded starting from the lowest standard. The analysis software was then used to fit a curve to this set of reliable standards data using five parameter logistic regression with default automated weighting (all fitted to ≥ 6 points). A similar standard curve optimisation process is now incorporated into the latest software release and was used for experiments to assess endogenous cytokines in clinical samples.

Lower and upper limits of quantification (LLOQ and ULOQ) were calculated as the highest and lowest measured reliable standards for each standard curve after optimisation as above. The linear dynamic range (LDR) was defined as the lowest and highest standards on the linear part of each standard curve on a log–log plot. Additional experimental readouts were spiked cytokine recovery (measure of accuracy, [observed concentration / expected concentration] × 100, acceptance criteria ± 20%), repeatability (measure of intra-assay precision, %CV, acceptance criteria < 25%) and total protein recovery using a bicinchoninic acid (BCA) assay kit (Pierce, IL, USA).

### 2.5 Real-time reverse transcriptase polymerase chain reaction (RT-qPCR)

Gastric biopsies were transferred at endoscopy to RNA*later* solution (Sigma-Aldrich) and preserved at − 80 °C. Total RNA was extracted after homogenisation with a TissueRuptor rotor–stator using an AllPrep DNA/RNA mini kit (QIAGEN). RNA concentration and purity were assessed using a NanoDrop ND-1000 spectrophotometer (NanoDrop Technologies, DE, USA) and integrity assessed using an Agilent 2100 Bioanalyzer microfluidic platform (Agilent Technologies, CA, USA). After DNase treatment with Ambion Turbo DNA-free kit (Applied Biosystems, CA, USA), cDNA was synthesised using SuperScript II reverse transcriptase with hexamer random primers (both Invitrogen, CA, USA). Quantification of mRNA transcripts of *IL17A*, *IFNG*, *IL8* and the reference gene *GAPDH* was performed using DyNAmo SYBR Green PCR master mix (Finnzymes, Thermo Fisher Scientific, MA, USA) on a Corbett Rotor Gene 3000 system (QIAGEN). Amplification was carried out in triplicate over 40 to 45 cycles of 15 s at 95 °C, 30 s at 61 °C (*IFNG*, *GAPDH*) or 62 °C (*IL17A*, *IL8*, *GAPDH*) and 30 s at 72 °C. Included in each assay were commercial human cDNA (Clontech, BD Biosciences, CA, USA) positive controls, no template controls and first-stage RT minus controls. Specificity analysis was performed with high resolution melt curves. Results were analysed by Pfaffl's relative quantification method (Pfaffl, 2001), normalising against *GAPDH* and comparing against a pooled negative comparator prepared from a further 14 uninfected donors. Commercial primers were used for *IL17A* and *IFNG* (SABiosciences, QIAGEN). *IL8* primers were F: 5′-CTCTTGGCAGCCTTCCTGA and R: 5′-AGTTCTTTAGCACTCCTTGGCA. *GAPDH* primers were as previously described (Robinson et al., 2008). Data were analysed with Rotor-Gene software (version 6.1, Corbett Research, UK).

### 2.6 Statistical analysis

Statistical analysis was performed using Prism 6.00 (GraphPad, Software CA, USA). Continuous variables were compared using non-parametric Mann–Whitney *U*-tests. Two-tailed *p* < 0.05 was considered significant.

## 3 Results

### 3.1 Sensitivity, standard curves and technical considerations

One of our objectives was to assess cytokines present at low concentrations and therefore the performance of the three Luminex kits in terms of their sensitivity and assay range. Standard curves provided by each manufacturer were run as recommended but extended to < 1.0 pg/mL to further assess kit sensitivity. As expected all kits performed well within the standard curve ranges recommended by each manufacturer (Table 1), although the Bio-Plex kit was less sensitive for IFNγ in our hands with a lower limit of quantification (LLOQ) of 8.1 pg/mL (vs 1.9 pg/mL lowest recommended standard). The VersaMAP kit had the lowest LLOQ for IFNγ (0.3 pg/mL) although the lowest recommended standard for this kit was 27.2 pg/mL. For IL-17, the Bio-Plex kit was most sensitive with a LLOQ of 1.3 pg/mL. Overall the MILLIPLEX kit performed closest to the specified product characteristics for both analytes. In addition though the upper limits of quantification (ULOQ) were highest with the Bio-Plex kit, the MILLIPLEX kit provided the broadest linear dynamic ranges.

Low bead counts for a particular well can reduce confidence in the reported median fluorescence intensity and hence the analyte concentration value interpolated from a standard curve. Manufacturers generally validate their assays with soluble materials such as sera, plasma and cell culture supernatants. We therefore assessed kit performance with our samples — clarified supernatants from disrupted and homogenised mucosal tissue. Low bead counts were more common with the VersaMAP kit in our hands (> 90% of samples on some runs and up to 1 in 3 standard/control wells). In contrast for the Bio-Plex and MILLIPLEX kits, low bead counts were not observed in any standard/control wells and in 11% and 1% of samples respectively. This may have been a result of greater median bead aggregation observed with this type of sample for the VersaMAP kit than for the Bio-Plex and MILLIPLEX kits (29% vs 11% and 12% respectively).

Even though each kit performed as specified and intended by the manufacturers, our aim was to quantify low concentrations of both IL-17 and IFNγ in tissue samples. Given our findings for sensitivity, standard curves and technical performance, only the Bio-Plex and MILLIPLEX kits were evaluated further.

### 3.2 Spiked cytokine recovery (accuracy)

Spiked cytokine recovery was used to measure the ability of each kit to accurately quantify recombinant cytokines in tissue homogenates. Nine biopsies each from three patients were individually prepared by manual disruption in extraction buffer (A). Supernatants from each patient were combined and split into aliquots. For each set of aliquots from a single patient, one was spiked with extraction buffer alone (“unspiked”) and two were spiked with known concentrations of both recombinant human IL-17 and IFNγ. Therefore we evaluated the ability of each of the kits to accurately measure cytokine spikes in mucosal tissue homogenates at lower and higher concentrations (1.5, 6, 50, 100 and 1000 pg/mL; for range of standard curves see Table 1).

**Fig. 1:**
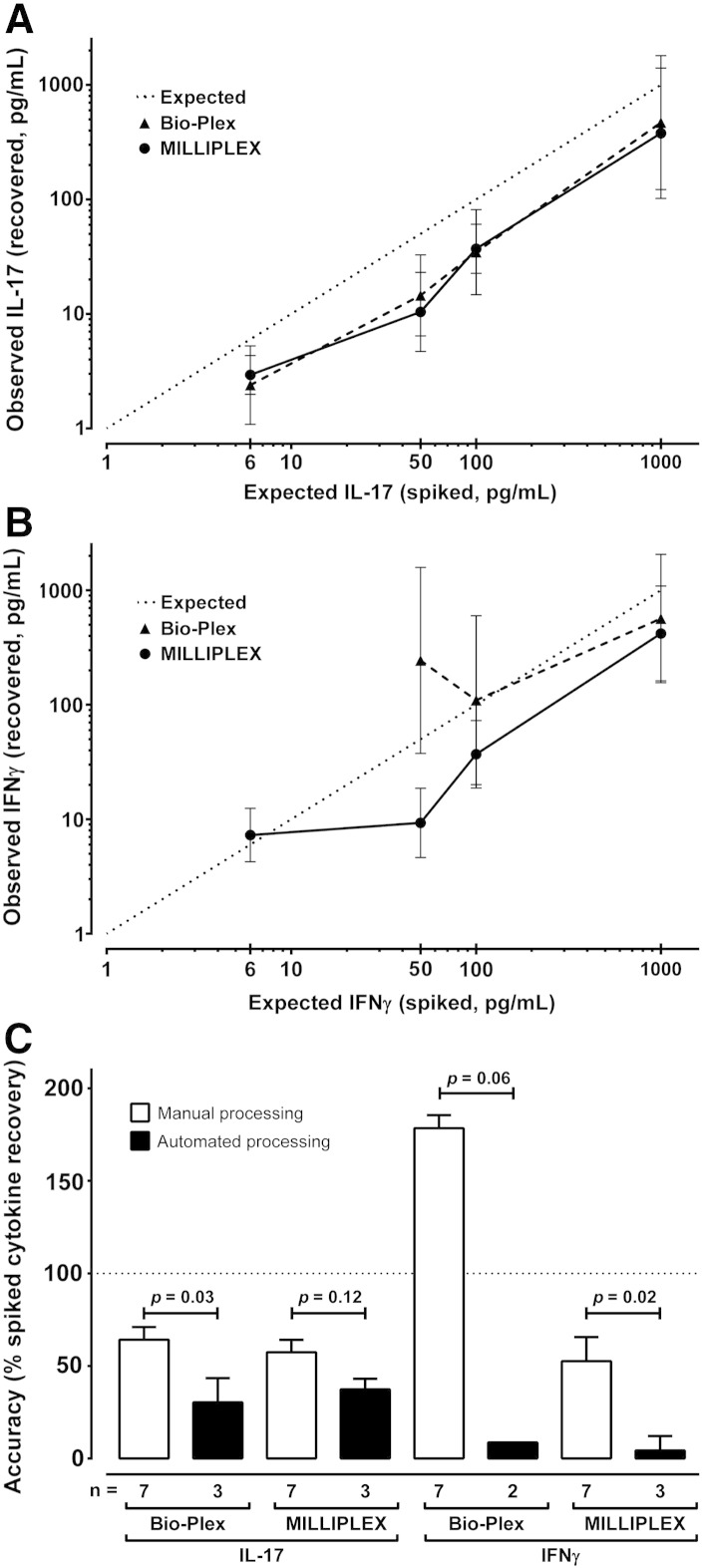
Accuracy of spiked cytokine recovery. A and B — Human gastric mucosal tissue homogenate supernatants were spiked with known concentrations of recombinant interleukin-17A (IL-17, panel A) and interferon-gamma (IFNγ, panel B), then cytokine concentrations assayed with different Luminex kits (Bio-Plex and MILLIPLEX, VersaMAP excluded). Data were adjusted for background using paired unspiked biopsies from the same patient, then expected and observed concentrations plotted on a log–log scale. Expected performance assuming 100% accuracy is shown by the dotted line. Error bars were calculated using the coefficient of variation (%CV) for each sample from all bead fluorescence intensities between 5th centile and 95th centile (trimmed bead %CV). No kits performed adequately < 6 pg/mL. Although it under-reports cytokine concentrations, the MILLIPLEX kit appears most consistent across the two analytes for spikes of 6–1000 pg/mL. C — We compared manual and automated tissue processing methods for four pairs of gastric mucosal biopsies from four patients. Manual methods included all biopsies disrupted with a pellet pestle with or without homogenisation using a needle and syringe. Automated processing using a bead-basher (TissueLyser LT, QIAGEN). Accuracy was calculated from percentage spiked cytokine recovery as [observed concentration / expected concentration] × 100. The figure shows median and inter-quartile range for each method, and comparisons using Mann–Whitney *U*-tests.

Observed IL-17 values were lower than expected for both the Bio-Plex kit (≥ 6 pg/mL: 38% ± 8% [mean ± SD], 29–47% [range]) and the MILLIPLEX kit (≥ 6 pg/mL: 36% ± 12%, 21–49%) — see Fig. 1A. Neither kit adequately measured IL-17 spike recovery at 1.5 pg/mL. The background levels in unspiked samples from the three patients were 0.0, 0.0 and 1.8 pg/mL for the Bio-Plex kit and slightly higher at 0.0, 2.4 and 2.5 pg/mL for the MILLIPLEX kit.

The IFNγ spikes were recovered with generally lower than expected accuracy using the MILLIPLEX kit (≥ 50 pg/mL: 32% ± 12%, 19–42%) and overall with higher than expected accuracy with the Bio-Plex kit (≥ 50 pg/mL: 218% ± 235%, 57–487%) — see Fig. 1B. Neither kit adequately measured IFNγ spike recovery at 1.5 pg/mL and only the MILLIPLEX kit performed as expected at 6 pg/mL (121%). High levels of IFNγ background were detected in the unspiked samples using the Bio-Plex kit (49.2, 264.0 and 1193.7 pg/mL) compared with background levels of 0.3, 4.5 and 6.7 pg/mL with the MILLIPLEX kit. Note that a control containing only the RPMI-1640 and FCS extraction buffer (A) yielded an IFNγ reading of 1177.7 pg/mL with the Bio-Plex kit compared with 0.0 pg/mL for the PBS-based extraction buffers (B) and (C). Since cytokine concentrations were to be normalised for biopsy total protein, we proceeded with serum-free media and therefore only extraction buffers (B) and (C) were used to assess repeatability and compare processing methods.

### 3.3 Repeatability (intra-assay precision)

We measured the precision of Bio-Plex and MILLIPLEX in quantifying spiked cytokine recovery across repeats of biological replicates within each individual assay, which we report as repeatability. Four identical aliquots of three different patient samples were included at different positions on the same plate. The coefficient of variation (%CV) was calculated for each sample and a mean %CV derived from the pooled %CV values. In this analysis the %CV was lower with the MILLIPLEX kit for IFNγ (15.4% vs 39.3%) and with the Bio-Plex kit for IL-17 (15.6% vs 21.7%).

We also measured the intra-assay precision of these two kits in quantifying cytokine concentrations derived from and included in standard curve calculations. The pooled mean %CV across all IL-17 standards was lower with the Bio-Plex kit (11.8% vs 24.2%) and across all IFNγ standards was lower with the MILLIPLEX kit (14.2% vs 25.1%). We have insufficient data to report on inter-assay precision.

### 3.4 Comparison of processing methods

Complex biological samples derived from tissues have not been evaluated by Luminex kit manufacturers and the optimal procedure to prepare our human mucosal tissue samples was not known. Determining the impact of different protocols on cytokine measures could improve the utility of Luminex-based methods to achieve our intended purpose — namely the quantification of endogenous cytokines present at low concentrations in small tissue samples. We compared processing methods and extraction buffers for four pairs of biopsies from each of four patients. Within each pair, biopsies were spiked at 100 pg/mL or spiked with buffer alone (“unspiked”), processed and then split into aliquots.

Manual sample disruption using a mini pellet pestle with or without homogenisation using a needle and syringe, and automated processing using a TissueLyser LT bead-basher (QIAGEN) were compared, as detailed in Materials and methods. Cytokine spikes were recovered significantly more accurately from samples processed manually (Fig. 1C). There were no significant differences between processing methods in relation to precision (data not shown) or total protein recovery by BCA assay (mean ± SD for manual 821.8 ± 108.0 μg/mL vs automated 800.3 ± 179.2 μg/mL).

We compared manual disruption using pestle alone with additional homogenisation using needle and syringe. Spiked cytokine recovery was usually lower with the latter (Table 2), although this difference was not consistent or statistically significant. We observed that homogenisation with a needle and syringe leads to loss of sample volume, which was retained in equipment dead space. In addition we evaluated if the addition of benzonase to PBS-based extraction buffer improved the performance of manual or automated processing. Benzonase is an endonuclease and digestion of nucleic acids may reduce sample viscosity. There was a consistent trend for increased cytokine recovery when benzonase was included in the extraction buffer but these differences did not reach statistical significance (Table 2).

### 3.5 Endogenous cytokines in clinical samples

To address the suitability of Luminex assays to detect endogenous cytokines in clinical samples we tested unspiked biopsies from uninfected and *Hp*-infected individuals using our final sample homogenisation protocol (see Section 4.3) for IL-17, IFNγ and also for IL-8, IL-4 and IL-10 using MILLIPLEX kits (see Section 2.4 and Section 4.2).

**Fig. 2:**
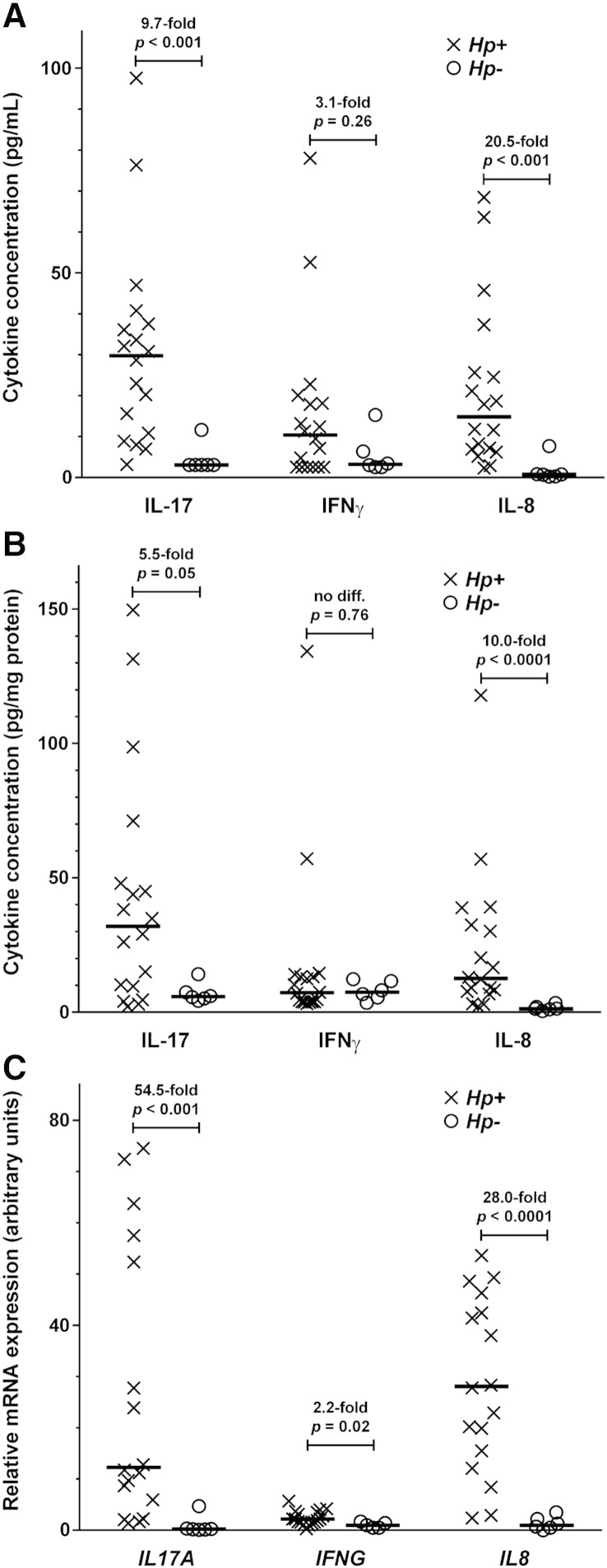
Endogenous cytokine expression at protein and mRNA levels in clinical samples. Levels of interleukin-17A (IL-17, *IL17A*), interferon-gamma (IFNγ, *IFNG*) and IL-8 (*IL8*) in unspiked antral human gastric tissue biopsies from patients infected with *Helicobacter pylori* (*Hp*+, X) and uninfected subjects (*Hp*−, O). The figures show each data point with horizontal bar for median value, fold-difference in medians between *Hp*+ and *Hp*−, and comparisons using Mann–Whitney tests. A and B — Cytokine concentrations were measured in clinical samples from 18 *Hp*+ and six *Hp*− subjects using our selected Luminex kit and optimised tissue processing method (see Sections 4.2 and 4.3), and reported unadjusted (panel A) and adjusted for total biopsy protein (panel B). C — Cytokine mRNA expression was determined using real-time reverse transcriptase polymerase chain reaction (RT-qPCR) in a further 41 consecutive patients such that each transcript was evaluated in 18 *Hp*+ and 6 *Hp*− patients as complete data were not available for every patient. Results were analysed by Pfaffl's relative quantification method (Pfaffl, 2001), normalising against *GAPDH* and comparing against a pooled negative comparator prepared from a further 14 uninfected donors. Note that for figure clarity two data points were not plotted — *IL17A Hp*+ 168.5 arbitrary units and *IL8 Hp*+ 111.6 arbitrary units.

We detected low background levels of IL-17, IFNγ and IL-8 in uninfected and uninflamed biopsies at or below the LLOQs for these analytes (2.8, 2.4 and 0.1 pg/mL respectively). However in *Hp*-infected biopsies there were marked 10 to 20 fold increases in IL-8 and IL-17 concentrations, and a smaller increase for IFNγ that did not reach statistical significance (Fig. 2A). These findings remained after correcting cytokine concentration for total biopsy protein (Fig. 2B). We were also able to detect differences in IL-10 in *Hp*-infected and uninfected tissues (median [inter-quartile range]; 10.0 pg/mg protein [8.4–15.0] and 1.3 pg/mg protein [1.1–4.0] respectively, *p* < 0.001, LLOQ 3.5 pg/mL) and to detect IL-4 (*Hp*+: 4.1 pg/mg protein [2.8–4.7], *Hp*−: 6.3 pg/mg protein [4.2–10.0], *p* = 0.08, LLOQ 2.9 pg/mL). Relative cytokine yield was comparable to mRNA expression quantified by RT-qPCR (Fig. 2C). The mean pooled intra-assay %CV across all reported analytes for standard curve cytokine measurements was 12.5% (7.3% for IL-17 and 12.1% for IFNγ).

## 4 Discussion

Our aim was the simultaneous quantification of multiple cytokines present in human mucosal biopsies, which are precious samples for translational researchers. Additional challenges were the limited tissue sample size and the low concentration of cytokines of interest in the healthy stomach. Multi-parameter Luminex assays are an attractive option but tissue samples are more complex than typical cell culture, plasma and sera samples with which these assays were developed. Ultimately our goal was an approach that would more accurately assess the in vivo cytokine profile. We evaluated the performance of three manufacturers' Luminex assays for IL-17 and IFNγ in human gastric biopsies spiked with recombinant cytokines and compared different approaches to sample preparation. We found that careful kit selection and sample preparation can improve the quality of data obtained from mucosal biopsies. Finally we assessed the suitability of our optimised approach for detecting endogenous cytokines.

### 4.1 Luminex kit performance — technical considerations and standard curves

We identified greater bead aggregation and consequently lower bead counts for the VersaMAP kit. This may in part be due to the different software settings used to classify beads as aggregates (DD gate). However the use of relatively viscous tissue homogenates and vacuum washing may retain sample matrix and clog the filter plate (Houser, 2012). Magnetic plate washing of paramagnetic Luminex beads may be an advantage for the analysis of tissue samples.

The MILLIPLEX kit had the advantage of requiring only 25 μL of sample per well, whereas for the VersaMAP and Bio-Plex kits the manufacturers recommended 50 μL of sample per well. In addition two further quality control vials were included with the MILLIPLEX kit with expected ranges, although these can only confirm standard curve integrity if reconstituted and measured in the same matrix as samples (Djoba Siawaya et al., 2008). The Bio-Plex kit was the fastest assay to perform with the longest incubation time of only 30 min. Both the VersaMAP and MILLIPLEX kits required incubations of 2 h after adding the samples then 1 h after adding the biotinylated detection antibody.

Each kit recommended a different dilution series for the standard curve: 3-fold 6-step for VersaMAP, 4-fold 8-step for Bio-Plex and 5-fold 6-step for MILLIPLEX. Therefore Luminex standard curves have a wider range than 2-fold dilutions for a typical ELISA standard curve. This maximises the number of wells available for samples and minimises the need to test/retest for multiple cytokines at different dilutions.

Finally it is important to consider analyte availability and compatibility in selecting kit(s) from a particular manufacturer.

### 4.2 Luminex kit performance — sensitivity, accuracy and precision

We found that assay sensitivity varied between manufacturers and analytes, as other authors have observed (Khan et al., 2004; duPont et al., 2005; Djoba Siawaya et al., 2008; Breen et al., 2011). The MILLIPLEX kit performed most consistently in our hands with a LLOQ ≤ 3.4 pg/mL and the broadest linear dynamic range for both IL-17 and IFNγ. No kits performed adequately with ≤ 1.5 pg/mL cytokine in spike recovery experiments. Greater sensitivity and resolution at the lower end of standard curves might be achievable by using the High RP1 target for instrument calibration or by adjusting the weighting of logistic regression curve fitting. Several manufacturers now market high-sensitivity/ultrasensitive Luminex kits, currently for a more limited number of analytes. These were recently investigated in a study of serum cytokine concentrations (Breen et al., 2011).

Accuracy of cytokine spike recovery frequently fell outside ± 25% of the expected values. However above the assay LLOQs the trend generally followed that of the expected values, even if the absolute values were different. Overall the MILLIPLEX kit performed most consistently over the widest range of spike concentrations, with spike recovery around one third of expected. Internal similarity in relative values but differences in absolute values have been noted in previous studies comparing different Luminex kits and Luminex kits with ELISA (Khan et al., 2004; Elshal and McCoy, 2006). In at least partial explanation, a study by Nechansky et al. (2008) compared cytokine standards from three commercial Luminex kits to WHO standards, and demonstrated discrepant concentrations in some instances, concluding that the assays were not fully quantitative. In addition although the samples, controls and standards were prepared with identical extraction buffer, the matrices differed as we did not, for example, pool multiple biopsy homogenates for addition to standards and controls. For researchers looking to report relative comparison of various samples within a single patient cohort and research centre, our approach may be acceptable provided that a single batch of identical standards is used. Breen et al. (2011) reached similar conclusions.

Our study identified imprecision as a potential important limitation of Luminex assays. Repeatability in this study showed high intra-assay %CV values (samples: 15–40%, standards: ≤ 25%) compared with some published data on Luminex kits (Biagini et al., 2004) but were consistent with others (Djoba Siawaya et al., 2008). This imprecision may in part be due to our repeated samples being closer to the LLOQ of each kit, as we were particularly interested in kit sensitivity. Subsequent evaluation of our final method showed improved intra-assay precision for standards (< 15%).

In summary, in our hands the MILLIPLEX kit delivered most consistent spiked cytokine recovery (35–50% accuracy), most consistent sensitivity at the lower limit of quantification, the greatest linear dynamic range, the lowest rates of bead aggregation and low bead counts, and the lowest sample volume requirements. We therefore selected MILLIPLEX kits for future studies, including high-sensitivity bead kits and use of magnetic plate washing. Interestingly Serelli-Lee et al. (2012) recently used MILLIPLEX assays to analyse mucosal cytokine levels in human gastric biopsies, although used traditional ELISA kits for IL-17 and IFNγ.

### 4.3 Optimisation of sample processing methods

We found that simple manual methods of disruption and homogenisation were consistently superior to automated methods with superior accuracy. This was unexpected but may be the result of sample loss across the relatively large surface area of the 5 mm beads used for automated processing or from cytokine degradation. However we also observed that homogenisation with a needle and syringe can lead to sample loss in equipment dead space, which can be avoided by aspiration into a pipette tip with similar orifice diameter. We were restrained by sample availability for optimisation (four pairs of biopsies each from four patients) so additional methodological variables could not be empirically evaluated. For example, a sonication-based approach would need detailed optimisation and, like rotor–stator homogenisation, has the disadvantages of sample heating and the need for larger extraction buffer volumes. We also avoided enzymatic, ionic detergent and chemical methods in anticipation of potential protein degradation and impacts on down-stream analysis. This is supported by our finding that commercial protein extraction kits were unsuitable, though others have used non-ionic detergents with success (Luzza et al., 2000; Newton et al., 2000).

When comparing cytokine concentrations in different gastric biopsies it is necessary to control for biopsy size, as opposed to comparisons of spike recovery from identical aliquots of supernatant. Some authors investigating cytokine concentrations in gastric biopsies have adjusted for biopsy weight (Serelli-Lee et al., 2012), whereas others have taken the approach of adjusting for total protein concentrations measured by either modified Lowry, Bradford or BCA assays (Crabtree et al., 1991; Yamaoka et al., 2001; Hwang et al., 2002; Shimizu et al., 2004; Queiroz et al., 2011). Similar to previous studies (Kusugami et al., 1999), the gastric biopsies were small with mean ± SD weight of 4.3 ± 2.9 mg (n = 18). Some researchers use clinical samples prepared for analysis immediately after collection (Yamaoka et al., 2001). However as our samples had been snap frozen they were associated with variable amounts of water and mucus during thawing, so weight was an unreliable measure of biopsy tissue content in our hands. Therefore we used total biopsy protein by BCA assay to normalise cytokine concentrations for biopsy size.

Optimisation of matrix/extraction buffer is also crucial for complex samples such as tissue homogenates, which Luminex kit manufacturers typically do not use when developing and validating their assays. We selected PBS-based extraction buffers without sera for our final method as we used BCA assays to measure total biopsy protein. There is precedent for the use of PBS-based buffers to assay cytokine concentrations by ELISA in human gastric biopsies (Yamaoka et al., 2001; Shimizu et al., 2004; Queiroz et al., 2011). We found a trend towards the addition of endonuclease to the extraction buffer increasing cytokine recovery though this did not reach statistical significance. Initially we also found high background readings for IFNγ with the Bio-Plex kit using the RPMI-1640 and FCS extraction buffer (A), and suspected that a component of the media may have interfered with the assay. However several studies have used similar matrices (duPont et al., 2005; Djoba Siawaya et al., 2008; Richens et al., 2010; Serelli-Lee et al., 2012). Some authors have reported matrix interaction effects leading to a high level of background in Luminex assays (Waterboer et al., 2006; Pickering et al., 2010). They overcame this using additives to suppress non-specific binding or by elimination of serum from their buffers and diluents.

Our final protocol after optimisation comprised: disruption in 300 μL of buffer (C) with a pellet pestle on ice, homogenisation by repeated aspiration into a 200 μL filter pipette tip (Axygen, CA, USA) to minimise volume loss, incubation on ice, centrifugation and division into aliquots for storage. One aliquot was used to quantify total protein by BCA assay.

### 4.4 Suitability for detection of endogenous cytokines

IL-17, IFNγ, IL-8, IL-4 and IL-10 were measured in unspiked gastric biopsies from 18 *Hp*-infected and six uninfected patients using our selected Luminex kit and optimised sample processing method to validate it for measurement of endogenous cytokines. We were able to detect low background levels of cytokines (with sensitivity of 0.1–3.5 pg/mL) and demonstrate an increased concentration of endogenous cytokines in disease, which were in keeping with mRNA expression data. These findings are consistent with published data on relative protein levels of these cytokines in *Hp*-infected and uninfected patients measured by ELISA, western blotting and Luminex in supernatants from gastric biopsy homogenate or gastric biopsy culture (Bodger et al., 1997; Luzza et al., 2000; Shimizu et al., 2004; Mizuno et al., 2005; Serelli-Lee et al., 2012).

## 5 Conclusions

Sensitive measurement of cytokine profiles using methodology that better reflects in vivo concentrations is technically challenging. Optimisation of processing methods can improve data acquisition from precious tissue samples. A number of factors need to be considered when selecting an assay, including the type and quantity of samples, the availability and multiplexing capabilities of the desired analytes, the expected range of concentrations and sensitivity required, specificity, accuracy, precision, time and cost. We selected Luminex assays from MILLIPLEX for use in future studies based on our evaluation findings. Together with our optimised sample preparation protocol we concluded that Luminex assays are a suitable technique for quantifying endogenous cytokines in mucosal biopsies. We hope that our approach will be more widely relevant for those seeking to quantify multiple cytokines in small tissue samples.

## Figures and Tables

**Table 1:** Lower limit of quantification (LLOQ), upper limit of quantification (ULOQ), linear dynamic range (LDR) and standard curves for the three kits tested for interleukin-17A (IL-17) and interferon-gamma (IFNγ). Standard curves shown are based on extended dilution series with figures in brackets showing lower and upper limits suggested by each manufacturer where these differ. LLOQ, ULOQ and LDR were determined as described in Materials and methods.

		LLOQ (pg/mL)	ULOQ (pg/mL)	LDR (pg/mL)		Standard curve (pg/mL)	
IL-17	VersaMAP	5.9	4643.2	5.9	4643.2	0.2 (19.0)	4616.0
	Bio-Plex	1.3	23,036.0	1.3	5120.4	0.3 (1.3)	21,505.0
	MILLIPLEX	3.4	8938.1	3.4	8938.1	0.2 (3.2)	9000.0 (10,000.0)
IFNγ	VersaMAP	0.3	5753.6	1.0	2397.6	0.3 (27.2)	6620.0
	Bio-Plex	8.1	30,659.5	26.5	7660.5	0.5 (1.9)	30,646.0
	MILLIPLEX	2.8	9143.7	2.8	9143.7	0.2 (3.2)	9000.0 (10,000.0)

**Table 2:** Comparison of different manual processing methods and addition of benzonase. Gastric mucosal biopsies were spiked with 100 pg/mL of interleukin-17A (IL-17) and interferon-gamma (IFNγ) prior to manual biopsy processing. Data were adjusted for background using paired unspiked biopsies from the same patient, and are equivalent to percentage spiked cytokine recovery. As described in Materials and methods, we compared disruption in phosphate-buffered saline (PBS)-based extraction buffer using a pellet pestle alone (n = 2), disruption with homogenisation with a needle and syringe (n = 3), and manual disruption/homogenisation in a PBS-based buffer which contained benzonase (n = 2).

		Median spiked cytokine recovery (pg/mL)		
Pestle		+	+	+
Needle/syringe			+	+
Benzonase				+
IL-17	Bio-Plex	56.0	50.8	67.6
	MILLIPLEX	52.3	41.9	61.9
IFN-γ	Bio-Plex	142.9	163.8	182.1
	MILLIPLEX	55.1	40.9	69.6
